# Physical and Mental Health of Caregivers and Educators of Preschool-Aged Children: Identifying Benefits and Barriers to Outdoor Time, How Outdoor Time Can Make a Difference for Health Equity, and Why Income Matters

**DOI:** 10.3390/ijerph22020236

**Published:** 2025-02-07

**Authors:** Amber L. Fyfe-Johnson, Carolyn J. Noonan, Maria B. Butcher, Magdalena K. Haakenstad

**Affiliations:** 1 Institute for Research and Education to Advance Community Health, Elson S. Floyd College of Medicine, Washington State University, Seattle, WA 98101, USA; carolyn.noonan@wsu.edu; 2 Elson S. Floyd College of Medicine, Washington State University, Spokane, WA 99202, USA; maria.butiu@wsu.edu; 3 Department of Urban Planning, University of Washington, Seattle, WA 98105, USA; mhaaken@uw.edu

**Keywords:** outdoor time, early childhood, social determinants of health, financial adversity, nature, equigenic

## Abstract

Outdoor time is positively associated with improved physical and mental health in adults. Little is known about the specific effects of outdoor time on health outcomes for parents and educators of preschool-aged children. Early childhood is a critical window for growth and development, as parental and educator stress negatively impacts young children; thus, it is of paramount importance to systematically support parents and educators during these developmental years. The objectives of this research were to use a cross-sectional natural experiment to (1) evaluate the association between outdoor time and physical and mental health in caregivers and educators who engage with preschool-aged children; (2) evaluate the association between income and physical and mental health in caregivers and educators who engage with preschool-aged children; and (3) identify benefits and barriers of outdoor time and the importance, availability, and accessibility of community resources for outdoor time. Participants were recruited from three stakeholder groups: preschool educators, parents of children attending an outdoor preschool, and parents of preschool-aged children in the local community. Participants completed a health needs assessment (*n* = 46) to assess demographics, mental and physical health outcomes, and benefits, barriers, and resources for outdoor time. Caregivers and educators in the higher income group (≥USD 70,000) were 41% (95% CI: 12%, 70%) more likely to report very good or excellent self-reported health. Mean anxiety, depression, and perceived stress were lower in the higher-income group. Caregivers and educators in the higher outdoor time group had lower body mass index (−5.5 kg/m^2^; 95% CI: −11.6, 0.7), and outdoor time appeared to be protective for general health independent of income. Thus, outdoor time may be a critical protective factor to enhance biological resilience for caregivers and educators, especially for those facing financial adversity.

## 1. Introduction

The health benefits of being in outdoor natural environments are well established in adults [1,2,3,4]. For children, outdoor environments can improve mental health [5,6,7,8], behavioral problems [9,10], and social and emotional development [11,12,13,14] and may promote lower BMI [15,16]. In one study of preschool-aged children from low-income families, higher neighborhood greenspace was associated with 12% lower obesity (95% CI: 0.79–0.99) than children from neighborhoods with lower greenspace [17]. Despite this robust and promising literature, little is known about the specific effects of outdoor time on health outcomes for caregivers and educators of preschool-aged children. Parent physical and mental health are the strongest predictors of child health [18,19,20,21]. Children 0–17 years (children 0–5 years were oversampled) with parents who report being in “excellent” or “very good” overall physical health have a 3.7 times higher odds of also having “excellent” or “very good” overall health than if their parents report lower levels of overall health after adjusting for other key demographic risk factors (e.g., income) [18]. According to the nationally representative National Survey of Children’s Health, promoting mental health in adult caregivers of children 0–17 years promotes mental health [19]. In a study specifically in parents of children 2.5–5 years, lower parent physical and mental health were both associated with more behavioral health problems in children [20]. Children (0–17 years) with caregivers reporting poor mental health had a reported 4.9 times higher risk of also having lower general health [19]. In addition, children of parents with higher incomes have improved general health in childhood and adulthood—for every additional USD 10,000 in parental income, the likelihood of ‘very good’ or ‘excellent’ health for that child in adulthood health increases by 4% [21]. Educator health also contributes to child health. Higher teacher psychological stress is associated with higher physiological stress (e.g., cortisol levels) in students [22] and lower academic achievement [23]. US educators are twice as likely to experience psychological stress and have lower wellbeing than other working adults [24]. Furthermore, early childhood is a critical window for growth and development, and parental and educator stress negatively impacts young children; thus, it is of paramount importance to systematically support parents and educators during these developmental years—for adults and children alike. Given the known benefits of outdoor time for adults and children alike, promoting outdoor time in educational settings offers promise to promote health in a population that is at high risk for burnout [24] and has strong potential to improve health. The intersection of caregiver, educator, and child health provides an unparalleled opportunity for both adults and children to benefit from research and interventions that promote caregiver and educator health.

Higher proportions of people of color and communities facing socioeconomic adversity experience inequities in access to parks and greenspaces [25,26,27,28,29]. Moreover, greenspaces accessible to under-resourced communities are smaller (6.4 vs. 14 acres) [30], more crowded [31], and lower quality [32] than those near adequately-resourced communities. Inequities in access to greenspaces disproportionately affect under-resourced families who stand to benefit from them the most [33,34,35]. Furthermore, contact with nature and greenspace may disproportionately benefit disadvantaged populations by attenuating the toxic effects of poverty—the “equigenic” effect [27,36,37,38,39,40,41,42]. Social determinants of health (SDoH), contextual factors in which a child lives, learns, and plays [43], are intractable risk factors for Early Life Adversity (ELA) [44,45,46]. Economic hardship is the most common SDoH for ELA [47,48], with 50% of US children living at or near poverty [49,50,51]. Nature-based schools offer a multi-level, systematic approach for childhood obesity prevention and management as they address four of the five SDoH Healthy People 2030 domains: (1) education access and quality (access to high quality early childhood education opportunities), (2) economic stability (access to free/reduced tuition), (3) neighborhood and built environment (access to greenspace and outdoor time), and (4) social and community context (social and community cohesion for children and parents with preschool-aged children) [43]. Great potential exists for obesity-prevention policies that improve equitable access for scalable, health-promoting neighborhood greenspaces that are accessible to the community [52,53], and promoting equitable access to nature for all children holds promise to narrow pediatric health inequities [54].

Given the substantial inequities to accessible, adequately sized, and high-quality greenspaces in historically marginalized communities [30,31,32], it is crucial to evaluate their importance, availability, and accessibility to inform interventions to address these inequities in under-resourced and historically marginalized communities. Urban planning of greenspaces has considerable potential to promote health in both individuals and communities by addressing existing environmental inequities [55]. Nonetheless, it is paramount that urban planning decision-making incorporates the needs and perspectives of the relevant communities, many of which may have cultural norms, values, and perspectives that are critical to ensuring that the greenspaces feel relevant to the community. Some communities may not have sufficient parks, while others may have sufficient park resources but lack adequate infrastructure to access the parks. Each community is different; thus, it is important to incorporate a community-based participatory research (CBPR) framework when addressing communities’ needs to promote nature-based exposures and outdoor time.

No study in any population has evaluated the effects of outdoor time for caregivers and educators of preschool-aged children nor examined the implications of financial adversity. The objectives of this research were to use a cross-sectional natural experiment to (1) evaluate the association between outdoor time and physical and mental health in caregivers and educators who engage with preschool-aged children; (2) evaluate the association between income and physical and mental health in caregivers and educators who engage with preschool-aged children; and (3) identify benefits and barriers of outdoor time and the importance, availability, and accessibility of community resources for outdoor time. We hypothesized that (1) above-median outdoor time would be associated with improved physical and mental health compared to below-median outdoor time for caregivers and educators who engage with preschool-aged children, (2) being adequately financially resourced would be associated with improved physical and mental health compared to being under-resourced for caregivers and educators who engage with preschool-aged children, and (3) adequately financially resourced respondents would identify more benefits and fewer barriers to outdoor time compared to under-resourced respondents, and the importance, availability, and accessibility of community resources for outdoor time would be higher for adequately resourced respondents. This study presents an ideal opportunity to better understand how outdoor time for caregivers and educators impacts health outcomes for adults through equitable and structural solutions in early childcare educational settings [56,57]. Great potential exists for policies that improve access to scalable, health-promoting, early childhood programs—for both adults and children [52,53]. Promoting equitable access to nature for all children holds promise to reduce health disparities, both in childhood and later into adulthood [54].

## 2. Materials and Methods

### 2.1. Theoretical Framework

Two theoretical frameworks informed this study: First, the National Institute on Minority Health and Health Disparities (NIMHD) framework uses a matrix that includes (1) four levels of influence (individual, interpersonal, community, and society), (2) five domains of influence (biological, behavioral, physical/built environment, sociocultural environment, and health care system), and (3) four health outcome domains (individual health, family/organizational health, community health, and population health) [58]. The NIMHD framework aligns closely with the familiar theoretical framework of social determinants of health (SDoH), contextual factors in which people are born, grow, work, live, and age [43,57] that directly affect health outcomes in adults and children alike. Second, we utilized a community-based participatory research (CBPR) framework [59] to ensure that the study was designed and implemented to meet the needs of the community, incorporate the voices and perspectives of the community, and serve the community to address health inequities. CBPR requires a substantial commitment to outreach in and with marginalized communities. Incorporating the perspectives of the community is critical to the fundamental premise of this framework, as research that prioritizes the community requires elevating cultural norms, values, and wisdom into the development of research questions and hypotheses, study design, and project implementation. An equity-based approach to research is advantageous not only for the prioritized communities but also for maintaining scientific integrity and an ethically sound commitment to CBPR. This philosophy is dedicated to promoting diversity, equity, inclusion, and accessibility in research.

### 2.2. Setting and Participants

This study was designed to address the opportunity gap that is perpetuated when under-resourced families and their children do not have equitable access to the outdoors. This work was informed by the historic and systemic lack of representation in outdoor natural environments of young children and families of color [25,26,28,29] and the accumulating research that outdoor time improves physical and mental health outcomes in childhood and adulthood [60]. This study was aligned with the Washington State Department of Children, Youth, and Families initiative for Washington to be the first state in the US to license outdoor preschools. Licensure was approved by the Washington State Senate in 2021 and allows the provision of state-level funding for free and reduced tuition and full-day programming, which offers unparalleled potential to improve access to early childhood education for families with limited resources. 

This study was approved by the Washington State University Institutional Review Board. All participants provided written informed consent. Participants were eligible to participate if they were (1) a current educator or administrator in an outdoor preschool, (2) a parent of a child attending an outdoor preschool, or (3) a parent of a preschool-aged child in the partnering community. Recruitment occurred in the first four calendar months of 2022 (January–April) using study flyers distributed at local community centers and early childhood education settings and the local early childhood community in King County, Washington. A USD 50 gift card was distributed to all participants.

### 2.3. Procedures and Application of Theoretical Framework

All participants completed a cross-sectional health needs assessment (*n* = 49) to assess demographics, mental and physical health outcomes, and benefits, barriers, and resources for outdoor time. The survey administration time was approximately 20 min. Surveys were administered using Research Electronic Data Capture (REDCap) [61,62]. The research team applied the NIMHD framework by incorporating the following: (1) two of the four levels of influence (individual and community), (2) four of the five domains of influence (biological, behavioral, physical/built environment, and sociocultural environment), and (3) one of the four health outcome domains (individual). The research team applied the CBPR framework using the following approaches: (1) deepening a long-standing relationship with community partners that was centered on addressing the needs of the community and generating results that would have a direct impact on the community, (2) leading recruitment and enrollment efforts to lessen the burden on community partners, (3) conducting all research activities in the community at times and locations that were convenient for individual participants, and (4) meeting with the community and discussing the results to ensure that interpretation and dissemination of results aligned with the community needs and expectations.

### 2.4. Measures 

#### 2.4.1. Demographic Variables

Demographic variables included age (years), racial identification (not Caucasian or Caucasian), sex assigned at birth (male/female), gender identification (male, female, transgender, or non-binary), employment status (out of work, unable to work, employed), highest terminal degree (e.g., high school, bachelor’s degree, etc.), and annual household income (e.g., <USD 70,000 and ≥USD 70,000). Income was dichotomized based on the approximate criteria for free or reduced tuition eligibility at licensed preschools in Washington state.

#### 2.4.2. Outdoor Time Exposure

Outdoor time was assessed using a self-reported question generated by the investigative team that asked how much time participants spent outside on weekdays and weekends (hours/minutes).

#### 2.4.3. Physical Health Outcomes

Physical health outcomes included self-reported general health (single-item question), sedentary time (watching television, sitting at a computer, reading, playing video games, or other activities that do not require much movement or physical activity), a brief medical history (current prediabetes, diabetes, and high blood pressure), current smoking status, and self-reported height (feet/inches) and weight (pounds). Height and weight were used to calculate body mass index (BMI) in kilograms/meters^2^.

#### 2.4.4. Mental Health Outcomes

Mental health outcomes included anxiety (General Anxiety Disorder-7 scale; scores can range from 0 to 21, and higher scores indicate more anxiety), depression (Patient Health Questionniare-9; question assessing suicidal ideation removed due to limited resources to support suicidality and suicidal ideation; scores can range from 0 to 24 and higher scores indicate more depression), psychological stress (Perceived Stress Scale; scale scores can range from 0 to 40 and higher scores indicate more stress), adverse childhood experiences, and resilience (Brief Resilience Scale; scale scores can range from 1 to 5, and higher scores indicate more resilience).

#### 2.4.5. Benefits and Barriers of Outdoor Time and Importance, Availability, and Accessibility of Community Resources for Outdoor Time

The benefits and barriers of outdoor time were assessed using self-generated questions developed by the study team in collaboration with our community partners. Community resources for outdoor time were assessed with an adapted National Institutes of Health PhenX toolkit instrument, Coping with COVID through Nature (CCN). The CCN was designed to compare pre-COVID and during-COVID nature exposure. For this study, only questions about current nature exposure were assessed. The CCN asks about the importance, availability, and accessibility of different natural resources for outdoor time in community settings (e.g., neighborhood parks, trails for hiking, etc.).

### 2.5. Data Analysis

Descriptive statistics were calculated using means, standard deviations, and ranges for continuous variables, and frequencies or counts for categorical variables. Linear regression models were used to evaluate the associations between exposures (income and outdoor time) and continuous outcomes of interest (BMI and mental health). Logistic regression and marginal standardization were used for dichotomous outcomes (general health). All models were adjusted for sociodemographic confounders (e.g., age, race, and income when appropriate). Results are presented as point estimates with 95% confidence intervals [63], and all statistical analyses were conducted using Stata (version 17 or later) [64]. Results are interpreted as aggregated based on the explicit request of our community partners, as this approach aligned with their values, priorities, and community needs to address health inequities. It was also a priority for our community partners to have data collected from the three key stakeholder groups identified (outdoor preschool educators and caregivers/parents of children attending outdoor preschool and indoor preschools). Disaggregated results are included in Supplemental Materials (Table S1).

## 3. Results

### 3.1. Descriptive Sociodemographic and Health Characteristics

Of the *n* = 49 participants enrolled, 47 (96%) completed the survey, and 46 (94%) completed the income item on the survey. *n* = 46 is the analytic sample. Eleven (24%) participants self-identified as racialized, and five (11%) self-identified as having Hispanic, Mexican, or Latino/Latina ethnicity. The study sample was overwhelmingly female (93%), and the mean age was 33.7 years old. In comparison to adequately resourced counterparts (≥USD 70,000 annual income), under-resourced participants were more likely to have a high school diploma/GED (28% vs. 4%) and less likely to have a post-graduate or professional degree (19% vs. 56%) as the highest terminal degree (Table 1). In comparison to adequately resourced counterparts (≥USD 70,000 annual income), under-resourced participants were more likely to report “poor” or “fair” health (19% vs. 8%), higher BMI (27.9 vs. 25.5), higher anxiety scores (7.3 vs. 5.6), depression scores (5.9 vs. 3.8), stress scores (16.2 vs. 14.7), ≥3 adverse childhood experiences (47% vs. 20%), and have ≥5 h of sedentary time per day (43% vs. 24%) (Table 1). The median outdoor time for the overall sample was 840 min per week. Outdoor time was higher for under-resourced participants (1860 min per week) compared to adequately resourced participants (480 min per week) (Table 1). Educator status and annual household income < USD 70,000 were highly correlated (76%), and all educators were employed by an outdoor preschool (Supplemental Materials, Table S1).

### 3.2. Association of Physical and Mental Health Constructs with Weekly Outdoor Time and Income

When comparing participants with above the median weekly outdoor time to participants with below the median weekly outdoor time, outdoor time was associated with 4% lower “very good” or “excellent” general health (95% CI: −37%, 30%) and 5.5 kg/m^2^ (95% CI: −11.6, 0.7) lower BMI after adjusting for age, race, and income. Mean anxiety, depression, and stress scores were all lower among those with higher compared to lower outdoor time, though the magnitude of these differences was small. No differences were found in mean resilience scores (Table 2).

When comparing participants with an annual household income of greater than or equal to USD 70,000 to less than USD 70,000, those that were adequately resourced had 41% higher “very good” or “excellent” general health (95% CI: 12%, 70%), 5.5 kg/m^2^ lower BMI (95% CI: −11, 0), and a 2.5 lower mean depression score (95% CI: −5.5, 0.5) after adjusting for age and race. Mean anxiety and stress were also lower in those who were adequately resourced compared to those under-resourced, but the magnitudes of these associations were lower and not statistically significant. No differences were found in mean resilience scores (Table 3).

### 3.3. Outdoor Time Benefits, Barriers, and Importance, Availability, and Accessibility of Community Resources for Outdoor Time

**Figure 1:**
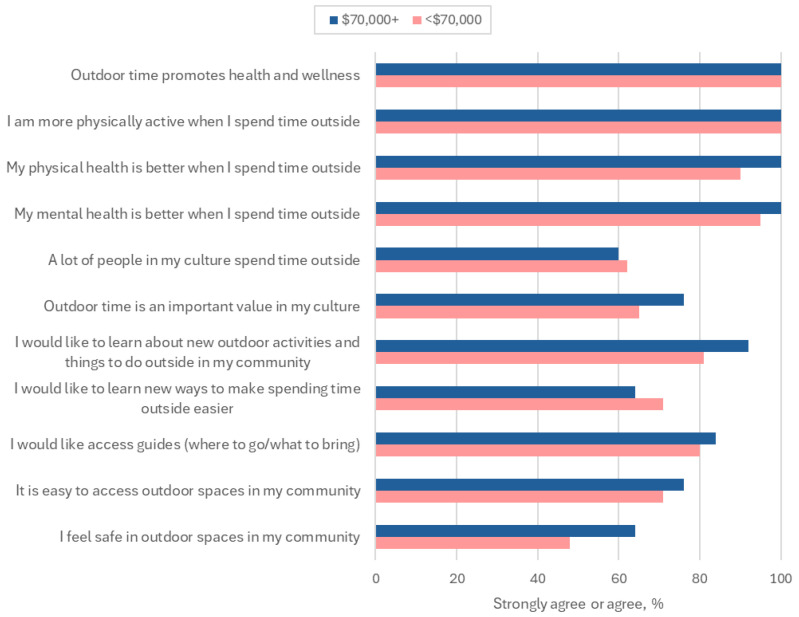
Outdoor time benefits and barriers according to annual household income.

When assessing the benefits of outdoor time, all participants universally reported that outdoor time promotes health and wellness and that they are more physically active when they are outside. The overwhelming majority of participants reported that their physical (95%) and mental health (98%) were better when they spent time outside (Figure 1; Supplemental Materials, Table S2). When assessing barriers to outdoor time, 48% of under-resourced participants and 64% of adequately resourced participants reported they agreed or strongly agreed that outdoor spaces in their community felt safe. The majority of under-resourced participants (81%) and adequately resourced participants (92%) reported they would like to learn about new outdoor activities and things to do outside in their community (Figure 1; Supplemental Materials, Table S2).

In both groups, most participants agreed or strongly agreed that a lot of people in their cultures spend time outside (62% under-resourced, 60% adequately resourced) and that outdoor time is an important value in their culture (65% under-resourced, 76% adequately resourced). Both groups reported wanting brief access guides for where to go and what to bring (80% under-resourced, 84% adequately resourced) (Figure 1; Supplemental Materials, Table S2).

**Figure 2:**
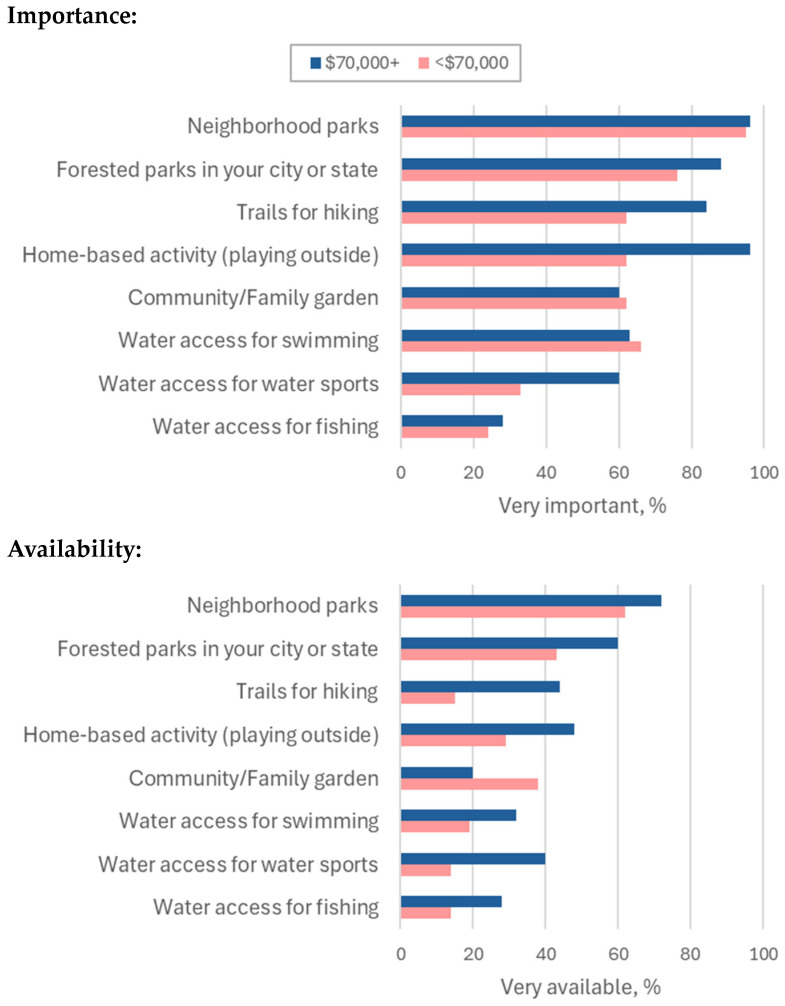

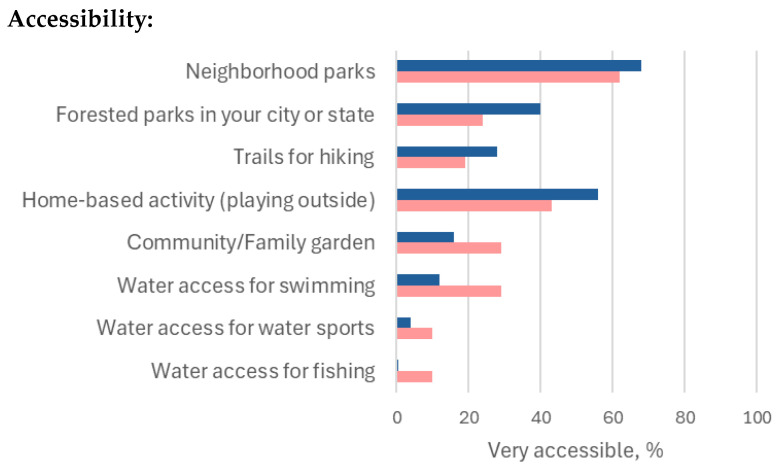
Importance, availability, and accessibility of outdoor resources according to annual household income.

In terms of the importance of outdoor time, both under-resourced and adequately resourced groups, the most important outdoor resources were neighborhood parks, city or state forested parks, trails for hiking, playing outside at home, and community or family gardens. Both groups reported that neighborhood parks are the most important outdoor community resource (95% under-resourced, 96% adequately resourced) and city or state parks were the next most important outdoor community resource (76% under-resourced, 88% adequately resourced) (Figure 2; Supplemental Materials, Table S3).

Regarding the availability of the outdoor resources deemed most important, three of the four categories were more available to adequately resourced respondents. There was a 10% (62% vs. 72%), 17% (43% vs. 60%), 29% (15% vs. 44%), and 19% (29% vs. 48%) lower availability for neighborhood parks (most important resource for both groups), city or state forested parks, trails for hiking, and outdoor time at home, respectively, for under-resourced respondents. There was an 18% higher availability of community or family gardens for under-resourced respondents (38% vs. 20%) compared to adequately resourced respondents (Figure 2; Supplemental Materials, Table S3).

Accessibility to outdoor community resources was similar between under-resourced and adequately resourced respondents for neighborhood parks (62% vs. 68%). Inequities in access were present between under-resourced and adequately resourced participants for city or state forested parks (24% vs. 40%), trails for hiking (19% vs. 28%), and outdoor time at home (43% vs. 56%) (Figure 2; Supplemental Materials, Table S3). Community or family gardens were more accessible to under-resourced respondents compared to adequately resourced respondents (29% vs. 16%). Across all outdoor resource categories, most respondents in both groups reported that most of the outdoor community resources were not easy to access (less than 50% reported that the outdoor resource was easy to access) (Figure 2; Supplemental Materials, Table S3).

## 4. Discussion

### 4.1. Outdoor Time and Physical and Mental Health Outcomes: Why Income Matters

Our results comparing adequately-resourced participants to under-resourced participants are consistent with previous literature that financial adversity is associated with poor physical and mental health outcomes [45,46,65]. It was important to replicate these results prior to conducting our first aim to ensure that the influence of financial adversity aligned with the current literature. It was unexpected that no differences were found in mean resilience scores (Table 3). After adjusting for age, race, and income, participants above the median of weekly outdoor time had both lower general health and lower BMI. It was unexpected and unclear why general health was lower, though the wide 95% confidence intervals suggest it to be due to the high variance within a small sample size. These BMI results are noteworthy because they suggest that outdoor time may be beneficial for health promotion, even for historically marginalized or under-resourced communities. While it is clear that high-quality early childhood education programs promote health equity in children [66] and outdoor time in childhood and adulthood benefits physical and mental health outcomes [60], these results lend further confidence that outdoor time—specifically in adults who engage with preschool-aged children—has the potential to promote health equity and may buffer the health deleterious effects of financial adversity. Given that children from under-resourced families and communities face steeper barriers to spending time in outdoor natural spaces, these results may also accelerate efforts to eliminate health inequities by improving universal access to greenspace for all people.

### 4.2. Outdoor Time Benefits and Barriers and Importance, Availability, and Accessibility of Community Resources for Outdoor Time

It is encouraging that all participants reported that outdoor time promotes health, wellness, and physical activity. These results contradict assumptions that under-resourced communities do not prioritize or value outdoor time [67,68,69]. These findings add to the overwhelming body of scientific literature supporting outdoor time for health promotion by adding a new perspective that people, independent of financial adversity, report that outdoor time is beneficial for health and wellness. The most influential barrier was concerns about safety when spending time outside. Simultaneously, participants in both groups expressed interest in learning about new outdoor activities in their communities, ways to make spending time outside easier, and access guides for outdoor spaces in their communities. This finding creates a rich opportunity for community organizations, leaders, and researchers to conduct outreach and educational activities to engage with people about new ways to spend time outside, both locally and broadly. This is especially relevant given that both groups reported the desire to have access to additional resources for where to go, what to do, and what to bring for outdoor activities. Future research is essential to identify and better understand the specific safety concerns in outdoor spaces and how to address these barriers for adults engaging with young children.

All groups reported that parks—neighborhood, city, and state—were the most important outdoor community resources. Neighborhood parks, in particular, were important. This is intuitive given that parents of preschool-aged children are often overwhelmed with the day-to-day logistics of navigating life and educators in particular are known to have high levels of burnout [24,34,70]. These results clarify that adults who engage with young children need to have available and accessible parks, especially in proximity to their neighborhoods. This aligns with the literature regarding travel barriers to accessing medical care and that people who experience more travel barriers face more challenges to accessing adequate medical care and thus have poorer health [71]. Similarly, if travel barriers to outdoor spaces such as parks are present, it will be more difficult and thus less likely for people to access, use, and experience the health benefits of these outdoor resources. This also aligns with the Trust for Public Land portfolio of work that aims to have all people have a park within a 10 min walk of their home [34,39]. According to the Trust for Public Land, one-third of US residents—100 million people and 28 million children—do not have access to a greenspace or park within a 10 min walk of their home [34]. This presents an unparalleled opportunity to synergize, promote, and accelerate work that is being done to promote accessible and available greenspace and parks, and the health benefits thereof, to millions of US residents.

It was expected that the outdoor resources deemed most important—neighborhood and city/state parks—were more available for adequately resourced respondents. These results align with current literature reporting that under-resourced communities experience inequities in access to parks and greenspaces [25,26,27,28,29]. Moreover, the accessible greenspaces are often less than half the size [30], more crowded [31], and have lower-quality natural spaces [32] than those near adequately resourced communities. These inequities often disproportionately affect under-resourced families, those who need them the most, given the toxic effects of poverty [27,36,37,38,39,40,41,42]. Our results suggest that availability was more of a barrier than accessibility based on resources (under-resourced respondents experienced less availability, on average, than those who were adequately resourced). Importantly, nearly all the community outdoor resources were not easy to access for all respondents. Accessibility appears to be a systemic barrier across the resource spectrum, whereas more barriers were present for the availability of outdoor resources for under-resourced people. These results provide important information that prioritizing parks at all levels (neighborhood, city, and state) may be worthwhile, given the importance of these outdoor resources.

For this study, all of the enrolled educators/administrators worked for nature-based schools, and some (but not all) of the enrolled parents had children attending outdoor preschools. This was an intentional study design decision, as our community partners were interested in the perspectives of parents of children attending outdoor and indoor programs and seasoned nature-based school educators/administrators. This community was in early conversations about expanding the availability of nature-based preschools to improve community health and was hoping to better understand the potential physical and mental health benefits for parents and other community members, as well as noteworthy barriers and benefits of outdoor time. While the results of this study were directly relevant to our community partners, an essential feature of CBPR, they may be informative for future policy work and urban planning initiatives to consider making outdoor spaces more inclusive [70], available in under-resourced communities, and more accessible for all communities [34].

### 4.3. Limitations and Strengths

The limitations of this study are fourfold. First, the analytic sample size is limited (*n =* 46); thus, our results are exploratory and hypothesis-generating in nature. Additional research with a more robust sample size would be beneficial for inferential results and broader generalizability of results. Second, all health outcomes were collected using self-report surveys administered electronically. Future research would benefit from collecting objectively measured physical health outcome data (e.g., objectively measured height and weight) and mental health outcome data (e.g., cortisol as a biomarker for stress). Third, the study was conducted in the Seattle metropolitan area from April to June 2022; thus, the study population is not nationally representative and has limited generalizability. Fourth, educator status and under-resourced status were highly correlated (76%). Thus, it is not possible to differentiate results by key stakeholder group (outdoor preschool parent, outdoor preschool educator, community parent with preschool-aged child). Lastly, BMI is a commonly used but imperfect method to measure adiposity because body fat is not directly assessed.

There are also numerous strengths to this study. First, the enrollment of participants in this study was supported by our early childhood education network community partners, which is critical to obtaining a community-informed perspective. The community partner’s support and research engagement enabled us to collect rich experiences and valuable insights into the topic from outdoor educators. Second, this work was informed by the historical and systemic lack of representation of young children and families of color in nature and the accumulating research that outdoor time improves physical health, development, and mental health outcomes in childhood. Third, this study brings innovative findings into the experiences and perspectives of early childhood caregivers and educators facing financial adversity, who are underrepresented in early childhood outdoor education research.

## 5. Conclusions

In conclusion, adequately resourced caregivers and educators who spent more time outside had improved physical and mental outcomes, independent of income. Outdoor time may be a critical protective factor to enhance physical and mental health equity and to promote biological resilience for caregivers and educators facing financial adversity [72]. Given that Washington was the first state to license outdoor preschools, these results are promising for the public health potential of changing state education policy to promote nature-based programming, outdoor time, and accessibility of nature-based outdoor spaces. Expanding the availability of nature-based preschools, or simply promoting more outdoor time and access to nature-based outdoor spaces for educators in traditional educational settings, may promote physical and mental health equity in communities who need it the most [72,73]. Parks, especially neighborhood parks, are important outdoor resources for adults who engage with preschool-aged children, likely due to ease and proximity. Availability of outdoor resources was more limited for people in under-resourced circumstances, and accessibility was not easy for most of the outdoor community resources for both under-resourced and adequately resourced groups. These results suggest that neighborhood parks may offer a clear mechanism to provide important and accessible greenspace and outdoor time. Providing resources and information about new ways to spend time outside presents a straightforward intervention approach for promoting outdoor time. If replicated, these results provide clarity on actionable next steps for policymakers and individuals involved in built environment planning to promote health and environmental equity [72,73,74,75].

## Figures and Tables

**Table 1:** Descriptive sociodemographic and health characteristics (*n* = 46).

	Annual Household Income			
	<USD 70,000 (*n =* 21)		USD 70,000+ (*n =* 25)	
Sociodemographic				
Age, *mean yrs* (*SD*)	30.6	(6.6)	36.2	(4.7)
Race, *n* (*%*)				
Racialized	5	(24%)	6	(25%)
Non-racialized	16	(76%)	18	(75%)
Hispanic, Mexican, or Latino/Latina ethnicity, *n* (*%*)	3	(14%)	2	(8%)
Sex assigned at birth, *n* (*%*)				
Male	2	(10%)	1	(4%)
Female	19	(90%)	24	(96%)
Gender identity, *n* (*%*)				
Male	2	(10%)	1	(4%)
Female	16	(76%)	23	(92%)
Transgender	0	(0%)	1	(4%)
Non-binary	3	(14%)	0	(0%)
Current employment status, *n* (*%*)				
Out of work	1	(5%)	8	(32%)
Unable to work	1	(5%)	0	(0%)
Employed	19	(90%)	17	(68%)
Completed education, *n* (*%*)				
High school/GED	6	(28%)	1	(4%)
Technical/vocational degree or Associate degree	1	(5%)	1	(4%)
College graduate (Bachelor’s degree)	10	(48%)	9	(36%)
Post-graduate degree or professional degree	4	(19%)	14	(56%)
Key stakeholder status, *n* (*%*)				
Outdoor educator	16	(76%)	3	(12%)
Caregiver	5	(24%)	22	(88%)
**Physical and mental health**				
Self-reported general health, *n* (*%*)				
Poor	1	(5%)	0	(0%)
Fair	3	(14%)	2	(8%)
Good	8	(38%)	3	(12%)
Very good	6	(29%)	15	(60%)
Excellent	3	(14%)	5	(20%)
Body mass index (kg/m^2^), *mean* (*SD*)	27.9	(10.6)	25.5	(6.0)
GAD-7 anxiety scale, *mean* (*SD*)	7.3	(5.3)	5.6	(4.3)
PHQ-8 depression scale, *mean* (*SD*)	5.9	(5.1)	3.8	(4.0)
Perceived stress scale, *mean* (*SD*)	16.2	(6.7)	14.7	(5.7)
Count of 11 adverse childhood experiences, *mean* (*SD*)	2.3	(1.6)	1.4	(1.3)
Count of 11 adverse childhood experiences, *n* (*%*)				
0	5	(24%)	7	(28%)
1	1	(5%)	7	(28%)
2	5	(24%)	6	(24%)
3	5	(24%)	3	(12%)
4	3	(14%)	2	(8%)
5	2	(9%)	0	(0%)
Brief resilience scale, *mean* (*SD*)	3.2	(0.7)	3.4	(0.7)
Sedentary time (hours/day), *n* (*%*)				
1 h or less	0	(0%)	3	(12%)
2–4 h	12	(57%)	16	(64%)
5–7 h	8	(38%)	5	(20%)
8–9 h	1	(5%)	1	(4%)
Sleep (hours/night), *n* (*%*)				
4–7 h	8	(38%)	12	(48%)
8 h	11	(52%)	13	(52%)
9 h or more	2	(10%)	0	(0%)
Prediabetes, *n* (*%*)	2	(10%)	2	(8%)
Diabetes, *n* (*%*)	0	(0%)	1	(4%)
High blood pressure, *n* (*%*)	2	(10%)	2	(8%)
Current smoking status, *n* (*%*)				
Not at all	20	(95%)	25	(100%)
Every day	1	(5%)	0	(0%)
Outdoor time/week, *median minutes* (*min*, *max*)	1860	(0, 2700)	480	(135, 2880)

**Table 2:** Association of physical and mental health constructs with weekly outdoor time among caregivers and educators of preschool-aged children.

Health Construct	Crude Diff ^1^ (95% CI)	Adjusted ^2^ Diff ^1^ (95% CI)
Very good or excellent self-reported health, *%*	−13% (−41%, 14%)	−4% (−37%, 30%)
Body mass index, *mean* kg/m^2^	−4.9 (−9.9, 0.1)	−5.5 (−11.6, 0.7)
GAD-7 anxiety, *mean score*	−0.2 (−3.1, 2.7)	−1.7 (−5.4, 2.1)
PHQ-8 depression, *mean score*	−0.4 (−3.2, 2.4)	−0.9 (−4.3, 2.5)
Perceived stress scale, *mean score*	−0.7 (−4.4, 3.0)	−1.1 (−5.9, 3.7)
Brief resilience scale, *mean score*	−0.2 (−0.6, 0.3)	0.0 (−0.6, 0.5)

^1^ Diff = Prevalence or mean difference comparing participants with above-median weekly outdoor time to participants with below-median weekly outdoor time. ^2^ Adjusted for age, race, and income; CI = confidence interval.

**Table 3:** Association of physical and mental health constructs with income among caregivers and educators of preschool-aged children.

Health construct	Crude Diff ^1^ (95% CI)	Model 2 ^2^ Diff ^1^ (95% CI)
Very good or excellent self-reported health, %	37% (11%, 63%)	41% (12%, 70%)
Body mass index, *mean* kg/m^2^	−2.4 (−7.5, 2.7)	−5.5 (−11.0, −0.0)
GAD-7 anxiety, *mean score*	−1.8 (−4.7, 1.1)	−1.2 (−4.6, 2.3)
PHQ-8 depression, *mean score*	−2.1 (−4.8, 0.7)	−2.5 (−5.5, 0.5)
Perceived stress scale, *mean score*	−1.5 (−5.2, 2.2)	−1.8 (−6.0, 2.5)
Brief resilience scale, *mean score*	0.2 (−0.3, 0.6)	0.0 (−0.5, 0.5)

^1^ Diff = Prevalence or mean difference comparing participants with an annual household income of ≥USD 70,000+ to those with <USD 70,000. ^2^ Adjusted for age and race; CI = confidence interval.

## Data Availability

The data presented in this study cannot be shared through commonly used data-sharing repositories. The consent did not address the broad sharing of participant data, nor the potential risks associated with broad data sharing of these types of data.

## Supplementary Materials

The following supporting information can be downloaded at: https://www.mdpi.com/article/10.3390/ijerph22020236/s1, Table S1. Descriptive sociodemographic and health characteristics according to study group (*n* = 46), Table S2. Outdoor time benefits and barriers (*n* = 46), Table S3. Importance, availability, and accessibility of outdoor community resources (*n* = 46).

## References

[1] Bratman G.N., Hamilton J.P., Hahn K.S., Daily G.C., Gross J.J. (2015). Nature experience reduces rumination and subgenual prefrontal cortex activation. Proc. Natl. Acad. Sci. USA.

[2] Bratman G., Daily G., Benjamin J., Gross J. (2015). The benefits of nature experience: Improved affect and cognition. Landsc. Urban Plan..

[3] Atchley R.A., Strayer D.L., Atchley P. (2012). Creativity in the Wild: Improving Creative Reasoning through Immersion in Natural Settings. PLoS ONE.

[4] Frumkin H., Bratman G.N., Breslow S.J., Cochran B., Kahn P.H., Lawler J.J., Levin P.S., Tandon P.S., Varanasi U., Wolf K.L. (2017). Nature Contact and Human Health: A Research Agenda. Environ. Health Perspect..

[5] Jackson R.J.J., Tester J., Henderson S.W. (2008). Environment Shapes Health, Including Children’s Mental Health. J. Am. Acad. Child Adolesc. Psychiatry.

[6] Wells N., Evans G. (2003). Nearby Nature: A buffer of life stress among rural children. Environ. Behav..

[7] Kuo F.E., Taylor A.F. (2004). A potential natural treatment for attention-deficit/hyperactivity disorder: Evidence from a national study. Am. J. Public Health.

[8] Amoly E., Dadvand P., Forns J., López-Vicente M., Basagaña X., Julvez J., Alvarez-Pedrerol M., Nieuwenhuijsen M.J., Sunyer J. (2014). Green and Blue Spaces and Behavioral Development in Barcelona Schoolchildren: The BREATHE Project. Environ. Health Perspect..

[9] Balseviciene B., Sinkariova L., Grazuleviciene R., Andrusaityte S., Uzdanaviciute I., Dedele A., Nieuwenhuijsen M. (2014). Impact of Residential Greenness on Preschool Children’s Emotional and Behavioral Problems. Int. J. Environ. Res. Public Health.

[10] Markevych I., Tiesler C.M.T., Fuertes E., Romanos M., Dadvand P., Nieuwenhuijsen M.J., Berdel D., Koletzko S., Heinrich J. (2014). Access to urban green spaces and behavioural problems in children: Results from the GINIplus and LISAplus studies. Environ. Int..

[11] Burdette H.L., Whitaker R.C. (2005). Resurrecting free play in young children: Looking beyond fitness and fatness to attention, affiliation, and affect. Arch. Pediatr. Adolesc. Med..

[12] Burdette H.L., Whitaker R.C., Daniels S.R. (2004). Parental report of outdoor playtime as a measure of physical activity in preschool-aged children. Arch. Pediatr. Adolesc. Med..

[13] Heerwagen J., Orians G. (2002). The Ecological World of Children. Children and Nature: Psychological, Sociocultural, and Evolutionary Investigations.

[14] Kellert S. (2012). Building for Life: Designing and Understanding the Human-Nature Connection.

[15] Bell J.F., Wilson J.S., Liu G.C. (2008). Neighborhood greenness and 2-year changes in body mass index of children and youth. Am. J. Prev. Med..

[16] Dadvand P., Villanueva C.M., Font-Ribera L., Martinez D., Basagaña X., Belmonte J., Vrijheid M., Gražulevičienė R., Kogevinas M., Nieuwenhuijsen M.J. (2014). Risks and Benefits of Green Spaces for Children: A Cross-Sectional Study of Associations with Sedentary Behavior, Obesity, Asthma, and Allergy. Environ. Health Perspect..

[17] Lovasi G.S., Schwartz-Soicher O., Quinn J.W., Berger D.K., Neckerman K.M., Jaslow R., Lee K.K., Rundle A. (2013). Neighborhood safety and green space as predictors of obesity among preschool children from low-income families in New York City. Prev. Med..

[18] The Health of Parents and Their Children: A Two-Generation Inquiry—Child Trends—ChildTrends. https://www.childtrends.org/publications/the-health-of-parents-and-their-children-a-two-generation-inquiry.

[19] Wolicki S.B., Bitsko R.H., Cree R.A., Danielson M.L., Ko J.Y., Warner L., Robinson L.R. (2021). Mental Health of Parents and Primary Caregivers by Sex and Associated Child Health Indicators. Advers. Resil. Sci..

[20] Poppert Cordts K.M., Wilson A.C., Riley A.R. (2020). More than Mental Health: Parent Physical Health and Early Childhood Behavior Problems. J. Dev. Behav. Pediatr..

[21] McInnis N. (2023). Long-term health effects of childhood parental income. Soc. Sci. Med..

[22] Oberle E., Schonert-Reichl K.A. (2016). Stress contagion in the classroom? The link between classroom teacher burnout and morning cortisol in elementary school students. Soc. Sci. Med..

[23] Madigan D.J., Kim L.E. (2021). Does teacher burnout affect students? A systematic review of its association with academic achievement and student-reported outcomes. Int. J. Educ. Res..

[24] Steiner E.D., Doan S., Woo A., Gittens A.D., Lawrence R.A., Berdie L., Wolfe R.L., Greer L., Schwartz H.L. (2022). Restoring Teacher and Principal Well-Being Is an Essential Step for Rebuilding Schools: Findings from the State of the American Teacher and State of the American Principal Surveys.

[25] Nesbitt L., Meitner M.J., Girling C., Sheppard S.R.J., Lu Y. (2019). Who has access to urban vegetation? A spatial analysis of distributional green equity in 10 US cities. Landsc. Urban Plan..

[26] Nesbitt L., Quinton J. (2023). Invited perspective: Natureis unfairly distributed in the united states-but that’s only part of the global green equity story. Environ. Health Perspect..

[27] Mitchell R., Popham F. (2008). Effect of exposure to natural environment on health inequalities: An observational population study. Lancet.

[28] Klompmaker J.O., Hart J.E., Bailey C.R., Browning M.H.E.M., Casey J.A., Hanley J.R., Minson C.T., Scott Ogletree S., Rigolon A., Laden F. (2023). Racial, ethnic, and socioeconomic disparities in multiple measures of blue and green spaces in the United States. Environ. Health Perspect..

[29] Subiza-Pérez M., García-Baquero G., Fernández-Somoano A., Riaño I., González L., Delgado-Saborit J.M., Guxens M., Fossati S., Vrijheid M., Fernandes A. (2023). Social inequalities, green and blue spaces and mental health in 6–12 years old children participating in the INMA cohort. Health Place.

[30] ParkScore Home: Trust for Public Land. https://www.tpl.org/parkscore.

[31] Dai D. (2011). Racial/ethnic and socioeconomic disparities in urban green space accessibility: Where to intervene?. Landsc. Urban Plan..

[32] Wolch J.R., Byrne J., Newell J.P. (2014). Urban green space, public health, and environmental justice: The challenge of making cities ‘just green enough’. Landsc. Urban Plan..

[33] National Public Radio Parks In Nonwhite Areas Are Half the Size of Ones in Majority-White Areas, Study Says. https://www.npr.org/2020/08/05/899356445/parks-in-nonwhite-areas-are-half-the-size-of-ones-in-majority-white-areas-study.

[34] The Heat Is On|The Trust for Public Land. https://www.tpl.org/the-heat-is-on.

[35] Casey J.A., James P., Cushing L., Jesdale B.M., Morello-Frosch R. (2017). Race, ethnicity, income concentration and 10-year change in urban greenness in the United States. Int. J. Environ. Res. Public Health.

[36] Hartig T. (2008). Green space, psychological restoration, and health inequality. Lancet.

[37] Mitchell R.J., Richardson E.A., Shortt N.K., Pearce J.R. (2015). Neighborhood Environments and Socioeconomic Inequalities in Mental Well-Being. Am. J. Prev. Med..

[38] Wheeler B.W., Lovell R., Higgins S.L., White M.P., Alcock I., Osborne N.J., Husk K., Sabel C.E., Depledge M.H. (2015). Beyond greenspace: An ecological study of population general health and indicators of natural environment type and quality. Int. J. Health Geogr..

[39] Browning M.H.E.M., Rigolon A. (2019). Could nature help children rise out of poverty? Green space and future earnings from a cohort in ten U.S. cities. Environ. Res..

[40] Rigolon A., Browning M.H.E.M., McAnirlin O., Yoon H. (2021). Green Space and Health Equity: A Systematic Review on the Potential of Green Space to Reduce Health Disparities. Int. J. Environ. Res. Public Health.

[41] Sikorska D., Łaszkiewicz E., Krauze K., Sikorski P. (2020). The role of informal green spaces in reducing inequalities in urban green space availability to children and seniors. Environ. Sci. Policy.

[42] Mears M., Brindley P., Jorgensen A., Maheswaran R. (2020). Population-level linkages between urban greenspace and health inequality: The case for using multiple indicators of neighbourhood greenspace. Health Place.

[43] Social Determinants of Health—Healthy People 2030. https://odphp.health.gov/healthypeople/priority-areas/social-determinants-health.

[44] Lopez M., Ruiz M.O., Rovnaghi C.R., Tam G.K.Y., Hiscox J., Gotlib I.H., Barr D.A., Carrion V.G., Anand K.J.S. (2021). The social ecology of childhood and early life adversity. Pediatr. Res..

[45] Bhutta Z.A., Bhavnani S., Betancourt T.S., Tomlinson M., Patel V. (2023). Adverse childhood experiences and lifelong health. Nat. Med..

[46] Shonkoff J.P., Garner A.S., Siegel B.S., Dobbins M.I., Earls M.F., McGuinn L., Pascoe J., Wood D.L., High P.C., Donoghue E. (2012). The lifelong effects of early childhood adversity and toxic stress. Pediatrics.

[47] Adversity in Early Childhood—Center for American Progress. https://www.americanprogress.org/article/adversity-early-childhood/.

[48] Blair C., Raver C.C., Granger D., Mills-Koonce R., Hibel L. (2011). Allostasis and allostatic load in the context of poverty in early childhood. Dev. Psychopathol..

[49] Pediatrics, Council on Community (2016). Poverty and child health in the United States. Pediatrics.

[50] Pascoe J.M., Wood D.L., Duffee J.H., Kuo A. (2016). Mediators and adverse effects of child poverty in the United States. Pediatrics.

[51] Hair N.L., Hanson J.L., Wolfe B.L., Pollak S.D. (2015). Association of Child Poverty, Brain Development, and Academic Achievement. JAMA Pediatr..

[52] Fiscella K., Kitzman H. (2009). Disparities in academic achievement and health: The intersection of child education and health policy. Pediatrics.

[53] Peterson J.W., Loeb S., Chamberlain L.J. (2018). The intersection of health and education to address school readiness of all children. Pediatrics.

[54] American Public Health Association Improving Health and Wellness Through Access to Nature. https://www.apha.org/policies-and-advocacy/public-health-policy-statements/policy-database/2014/07/08/09/18/improving-health-and-wellness-through-access-to-nature.

[55] Lee A.C.K., Jordan H.C., Horsley J. (2015). Value of urban green spaces in promoting healthy living and wellbeing: Prospects for planning. Risk Manag. Healthc. Policy.

[56] Kumanyika S. (2017). Getting to Equity in Obesity Prevention: A New Framework.

[57] Trust for America’s Health State of Obesity 2024: Better Policies for a Healthier America. https://www.tfah.org/report-details/state-of-obesity-2024/.

[58] National Institutes of Health National Institute for Minority Health and Health Disparities Research Framework. https://www.nimhd.nih.gov/about/overview/research-framework/nimhd-framework.html.

[59] National Institutes of Health National Institute for Minority Health and Health Disparities Community-Based Participatory Research Program (CBPR). https://www.nimhd.nih.gov/programs/extramural/community-based-participatory.html.

[60] Fyfe-Johnson A.L., Hazlehurst M.F., Perrins S.P., Bratman G.N., Thomas R., Garrett K.A., Hafferty K.R., Cullaz T.M., Marcuse E.K., Tandon P.S. (2021). Nature and children’s health: A systematic review. Pediatrics.

[61] Harris P.A., Taylor R., Thielke R., Payne J., Gonzalez N., Conde J.G. (2009). Research electronic data capture (REDCap)—A metadata-driven methodology and workflow process for providing translational research informatics support. J. Biomed. Inform..

[62] Harris P.A., Taylor R., Minor B.L., Elliott V., Fernandez M., Neal L.O., Mcleod L., Delacqua G., Delacqua F., Duda S.N. (2019). The REDCap consortium: Building an international community of software platform partners. J. Biomed. Inform..

[63] Greenland S., Senn S.J., Rothman K.J., Carlin J.B., Poole C., Goodman S.N., Altman D.G. (2016). Statistical tests, P values, confidence intervals, and power: A guide to misinterpretations. Eur. J. Epidemiol..

[64] Stata Statistical Software for Data Science. https://www.stata.com/.

[65] Shonkoff J.P., Richter L., Van Der Gaag J., Bhutta Z.A. (2012). An integrated scientific framework for child survival and early childhood development. Pediatrics.

[66] Hahn R.A., Barnett W.S., Knopf J.A., Truman B.I., Johnson R.L., Fielding J.E., Muntaner C., Jones C.P., Fullilove M.T., Hunt P.C. (2016). Early Childhood Education to Promote Health Equity. J. Public Health Manag. Pract..

[67] Center for American Progress The Nature Gap. https://www.americanprogress.org/article/the-nature-gap/.

[68] North Carolina State University, College of Natural Resources Nature Gap: Why Outdoor Spaces Lack Diversity and Inclusion. https://cnr.ncsu.edu/news/2020/12/outdoor-diversity-inclusion/.

[69] Sierra Club Why People of Color Often Feel Unsafe in the Outdoors. https://www.sierraclub.org/sierra/why-people-color-often-feel-unsafe-outdoors.

[70] The Atlantic Five Ways to Make the Outdoors More Inclusive. https://www.theatlantic.com/sponsored/rei-2018/five-ways-to-make-the-outdoors-more-inclusive/3019/.

[71] Syed S.T., Gerber B.S., Sharp L.K. (2013). Traveling towards disease: Transportation barriers to health care access. J. Community Health.

[72] Yan L., Jin X., Zhang J. (2024). Equity in park green spaces: A bibliometric analysis and systematic literature review from 2014-2023. Front. Environ. Sci..

[73] Public Health Institute Changing the Story About Park and Green Space Equity: A Messaging Guide for Advocates. https://www.phi.org/thought-leadership/changing-the-story-about-park-and-green-space-equity-a-messaging-guide-for-advocates/.

[74] Vox The American Outdoors Is Financially Inaccessible. Can it be More Equitable?. https://www.vox.com/23894176/great-outdoors-exclusionary-nature-hiking-equity.

[75] Network for Public Health Law The Great American Outdoors Act: A Tool to Advance Public Health. https://www.networkforphl.org/news-insights/the-great-american-outdoors-act-a-tool-to-advance-public-health/.

