# Supplementary material for: Physical and Mental Health of Caregivers and Educators of Preschool-Aged Children: Identifying Benefits and Barriers to Outdoor Time, How Outdoor Time Can Make a Difference for Health Equity, and Why Income Matters
Source: Int J Environ Res Public Health. 2025 Feb 7;22(2):236. doi: 10.3390/ijerph22020236 (PMC11855737; doi:10.3390/ijerph22020236)
Supplement: Supplementary file 1 [file ijerph-22-00236-s001.zip › ijerph-3346353-supplementary.docx]

**Supplemental Materials:**

| **Table S1.** Descriptive sociodemographic and health characteristics according to study group (n=46). | | | |
| --- | --- | --- | --- |
|  | **Study group** | | |
|  | **Outdoor preschool**  **parent**  **(n=13)** | **Outdoor preschool educator (n=19)** | **Community parent**  **(n=14)** |
| **Sociodemographic** |  |  |  |
| **Age**, *mean yrs (SD)* | 36.5 (3.5) | 29.3 (6.6) | 36.8 (4.4) |
| **Race**, *n (%)* |  |  |  |
| Racialized | 6 (47%) | 2 (11%) | 3 (23%) |
| Non-racialized | 7 (54%) | 17 (90%) | 10 (77%) |
| **Hispanic, Mexican, or Latino/Latina ethnicity**, *n (%)* | 0 (0%) | 3 (16%) | 2 (14%) |
| **Sex assigned at birth**, *n (%)* |  |  |  |
| Male | 1 (8%) | 2 (11%) | 0 (0%) |
| Female | 12 (92%) | 17 (89%) | 14 (100%) |
| **Gender identity**, *n (%)* |  |  |  |
| Male | 1 (8%) | 2 (11%) | 0 (0%) |
| Female | 11 (85%) | 14 (74%) | 14 (100%) |
| Transgender | 1 (8%) | 0 (0%) | 0 (0%) |
| Non-binary | 0 (0%) | 3 (16%) | 0 (0%) |
| **Current employment status**, *n (%)* |  |  |  |
| Out of work | 4 (31%) | 0 (0%) | 5 (36%) |
| Unable to work | 0 (0%) | 0 (0%) | 1 (7%) |
| Employed | 9 (69%) | 19 (100%) | 8 (57%) |
| **Completed education**, *n (%)* |  |  |  |
| High school/GED | 2 (15%) | 3 (16%) | 2 (14%) |
| Technical/vocational degree or Associate degree | 0 (0%) | 1 (5%) | 1 (7%) |
| College graduate (Bachelor’s degree) | 5 (39%) | 10 (53%) | 4 (29%) |
| Post-graduate degree or professional degree | 6 (46%) | 5 (26%) | 7 (50%) |
| **Annual household income**, *n (%)* |  |  |  |
| <$70,000 | 2 (15%) | 16 (84%) | 3 (21%) |
| $70,000+ | 11 (85%) | 3 (16%) | 11 (79%) |
| **Physical and mental health** |  |  |  |
| **Self-reported general health**, *n (%)* |  |  |  |
| Poor | 1 (8%) | 0 (0%) | 0 (0%) |
| Fair | 0 (0%) | 2 (11%) | 3 (21%) |
| Good | 0 (0%) | 8 (42%) | 3 (21%) |
| Very good | 10 (77%) | 7 (37%) | 4 (29%) |
| Excellent | 2 (15%) | 2 (11%) | 4 (29%) |
| **Body mass index (kg/m2),** *mean (SD)* | 29.6 (9.5) | 24.0 (5.2) | 27.5 (10.5) |
| **GAD-7 anxiety scale**, *mean (SD)* | 5.0 (3.6) | 7.1 (4.4) | 6.7 (6.1) |
| **PHQ-8 depression scale**, *mean (SD)* | 4.1 (3.6) | 5.0 (3.2) | 5.1 (6.9) |
| **Perceived stress scale**, *mean (SD)* | 14.7 (7.2) | 15.6 (4.3) | 15.6 (7.6) |
| **Count of 11 adverse childhood experiences**, *mean (SD)* | 1.5 (1.5) | 2.3 (1.6) | 1.6 (1.3) |
| **Count of 11 adverse childhood experiences,** *n (%)* |  |  |  |
| 0 | 5 (39%) | 4 (21%) | 3 (21%) |
| 1 | 2 (15%) | 1 (5%) | 5 (36%) |
| 2 | 3 (23%) | 6 (32%) | 2 (14%) |
| 3 | 1 (8%) | 4 (21%) | 3 (21%) |
| 4 | 2 (15%) | 2 (11%) | 1 (7%) |
| 5 | 0 (0%) | 2 (11%) | 0 (0%) |
| **Brief resilience scale**, *mean (SD)* | 3.5 (0.6) | 3.1 (0.6) | 3.5 (0.8) |
| **Sedentary time (hours/day)**, *n (%)* |  |  |  |
| 1 hour or less | 0 (0%) | 0 (0%) | 3 (21%) |
| 2 - 4 hours | 8 (62%) | 12 (63%) | 8 (57%) |
| 5 - 7 hours | 4 (31%) | 7 (37%) | 2 (14%) |
| 8 - 9 hours | 1 (8%) | 0 (0%) | 1 (7%) |
| **Sleep (hours/night)**, *n (%)* |  |  |  |
| 4 - 7 hours | 5 (39%) | 7 (37%) | 8 (57%) |
| 8 hours | 7 (54%) | 11 (58%) | 6 (43%) |
| 9 hours or more | 1 (8%) | 1 (5%) | 0 (0%) |
| **Pre-diabetes**, *n (%)* | 3 (23%) | 1 (5%) | 0 (0%) |
| **Diabetes**, *n (%)* | 1 (8%) | 0 (0%) | 0 (0%) |
| **High blood pressure**, *n (%)* | 1 (8%) | 2 (11%) | 1 (8%) |
| **Current smoking status**, *n (%)* |  |  |  |
| Not at all | 13 (100%) | 19 (100%) | 13 (93%) |
| Every day | 0 (0%) | 0 (0%) | 1 (7%) |
| **Outdoor time/week**, *median minutes (min, max)* | 675 (220, 1440) | 1907  (660, 2880) | 421 (0, 900) |

| **Table S2.** Outdoor time benefits and barriers (n=46). | | | | |
| --- | --- | --- | --- | --- |
|  | **Annual household income** | | | |
|  | **<$70,000 (n=21)** | | **$70,000+ (n=25)** | |
| **Outdoor time promotes health and wellness**, *n (%)* |  |  |  |  |
| Agree/Strongly Agree | 21 | (100%) | 25 | (100%) |
| **I am more physically active when I spend time outside**, *n (%)* |  |  |  |  |
| Agree/Strongly Agree | 21 | (100%) | 25 | (100%) |
| **My physical health is better when I spend time outside**, *n (%)* |  |  |  |  |
| Neutral | 2 | (10%) | 0 | (0%) |
| Agree/Strongly Agree | 19 | (90%) | 25 | (100%) |
| **My mental health is better when I spend time outside**, *n (%)* |  |  |  |  |
| Neutral | 1 | (5%) | 0 | (0%) |
| Agree/Strongly Agree | 20 | (95%) | 25 | (100%) |
| **I would like to learn new ways to make spending time outside easier**, *n (%)* |  |  |  |  |
| Strongly disagree/Disagree | 2 | (10%) | 4 | (16%) |
| Neutral | 4 | (19%) | 5 | (20%) |
| Agree/Strongly Agree | 15 | (71%) | 16 | (64%) |
| **A lot of people in my culture spend time outside**, *n (%)* |  |  |  |  |
| Strongly disagree/Disagree | 2 | (9%) | 3 | (12) |
| Neutral | 6 | (29%) | 7 | (28%) |
| Agree/Strongly Agree | 13 | (62%) | 15 | (60%) |
| **Outdoor time is an important value in my culture**, *n (%)* |  |  |  |  |
| Strongly disagree/Disagree | 2 | (15%) | 3 | (12%) |
| Neutral | 4 | (20%) | 3 | (12%) |
| Agree/Strongly Agree | 13 | (65%) | 19 | (76%) |
| **It is easy to access outdoor spaces in my community**, *n (%)* |  |  |  |  |
| Strongly disagree/Disagree | 2 | (10%) | 1 | (4%) |
| Neutral | 4 | (19%) | 5 | (20%) |
| Agree/Strongly Agree | 15 | (71%) | 19 | (76%) |
| **I feel safe in outdoor spaces in my community**, *n (%)* |  |  |  |  |
| Strongly disagree/Disagree | 2 | (9%) | 3 | (12%) |
| Neutral | 9 | (43%) | 6 | (24%) |
| Agree/Strongly Agree | 10 | (48%) | 16 | (64%) |
| **I would like to learn about new outdoor activities and things to do outside in my community**, *n (%)* |  |  |  |  |
| Strongly disagree/Disagree | 0 | (0%) | 1 | (4%) |
| Neutral | 4 | (19%) | 1 | (4%) |
| Agree/Strongly Agree | 17 | (81%) | 23 | (92%) |
| **I would like access guides (where to go/what to bring)**, *n (%)* |  |  |  |  |
| Strongly disagree/Disagree | 2 | (10%) | 0 | (0%) |
| Neutral | 2 | (10%) | 4 | (16%) |
| Agree/Strongly Agree | 16 | (80%) | 21 | (84%) |

| **Table S3.** Importance, availability, and accessibility of outdoor community resources (n=46). | | | | |
| --- | --- | --- | --- | --- |
|  | **Annual household income** | | | |
|  | **<$70,000 (n=21)** | | **$70,000+ (n=25)** | |
| **Importance of resources** | **1** | **0** | **1** | **0** |
| **Neighborhood parks**, *n (%)* |  |  |  |  |
| Somewhat important | 1 | (5%) | 1 | (4%) |
| Very important | 20 | (95%) | 24 | (96%) |
| **Forested parks in your city or state**, *n (%)* |  |  |  |  |
| Somewhat important | 5 | (24%) | 3 | (12%) |
| Very important | 16 | (76%) | 22 | (88%) |
| **Trails for hiking**, *n (%)* |  |  |  |  |
| Somewhat important | 8 | (38%) | 4 | (16%) |
| Very important | 13 | (62%) | 21 | (84%) |
| **Home-based activity (playing outside)**, *n (%)* |  |  |  |  |
| Not/Not very important | 1 | (5%) | 0 | (0%) |
| Somewhat important | 7 | (33%) | 1 | (4%) |
| Very important | 13 | (62%) | 24 | (96%) |
| **Community/family garden**, *n (%)* |  |  |  |  |
| Somewhat important | 8 | (38%) | 10 | (40%) |
| Very important | 13 | (62%) | 15 | (60%) |
| **Water access for swimming**, *n (%)* |  |  |  |  |
| Not/Not very important | 2 | (9%) | 1 | (4%) |
| Somewhat important | 5 | (24%) | 8 | (33%) |
| Very important | 14 | (66%) | 15 | (63%) |
| **Water access for water sports**, *n (%)* |  |  |  |  |
| Not/Not very important | 7 | (33%) | 2 | (8%) |
| Somewhat important | 7 | (33%) | 8 | (32%) |
| Very important | 7 | (33%) | 15 | (60%) |
| **Water access for fishing**, *n (%)* |  |  |  |  |
| Not/Not very important | 4 | (19%) | 12 | (48%) |
| Somewhat important | 12 | (57%) | 6 | (24%) |
| Very important | 5 | (24%) | 7 | (28%) |
| **Availability of resources for outdoor time** | *1* | *0* | *1* | *0* |
| **Neighborhood parks**, *n (%)* |  |  |  |  |
| A little available | 4 | (19%) | 2 | (8%) |
| Somewhat available | 4 | (19%) | 5 | (20%) |
| Very available | 13 | (62%) | 18 | (72%) |
| **Forested parks in your city or state**, *n (%)* |  |  |  |  |
| A little available | 4 | (19%) | 3 | (12%) |
| Somewhat available | 8 | (38%) | 7 | (28%) |
| Very available | 9 | (43%) | 15 | (60%) |
| **Trails for hiking**, *n (%)* |  |  |  |  |
| Not available | 1 | (5%) | 0 | (0%) |
| A little available | 5 | (25%) | 3 | (12%) |
| Somewhat available | 11 | (55%) | 11 | (44%) |
| Very available | 3 | (15%) | 11 | (44%) |
| **Home-based activity (playing outside)**, *n (%)* |  |  |  |  |
| Not available | 1 | (5%) | 0 | (0%) |
| A little available | 3 | (14%) | 3 | (12%) |
| Somewhat available | 11 | (52%) | 10 | (40%) |
| Very available | 6 | (29%) | 12 | (48%) |
| **Community/family garden**, *n (%)* |  |  |  |  |
| Not available | 2 | (10%) | 1 | (4%) |
| A little available | 6 | (29%) | 3 | (12%) |
| Somewhat available | 5 | (23%) | 16 | (64%) |
| Very available | 8 | (38%) | 5 | (20%) |
| **Water access for swimming**, *n (%)* |  |  |  |  |
| Not available | 2 | (10%) | 1 | (4%) |
| A little available | 7 | (33%) | 7 | (28%) |
| Somewhat available | 8 | (38%) | 9 | (36%) |
| Very available | 4 | (19%) | 8 | (32%) |
| **Water access for water sports**, *n (%)* |  |  |  |  |
| Not available | 2 | (10%) | 1 | (4%) |
| A little available | 7 | (33%) | 5 | (20%) |
| Somewhat available | 9 | (43%) | 9 | (36%) |
| Very available | 3 | (14%) | 10 | (40%) |
| **Water access for fishing**, *n (%)* |  |  |  |  |
| Not available | 3 | (14%) | 1 | (4%) |
| A little available | 10 | (48%) | 7 | (28%) |
| Somewhat available | 5 | (24%) | 10 | (40%) |
| Very available | 3 | (14%) | 7 | (28%) |
| **Accessibility of resources for outdoor time** | *1* | *0* | *1* | *0* |
| **Neighborhood parks**, *n (%)* |  |  |  |  |
| Difficult to access | 2 | (9%) | 1 | (4%) |
| Somewhat easy to access | 6 | (29%) | 7 | (28%) |
| Easy to access | 13 | (62%) | 17 | (68%) |
| **Forested parks in your city or state**, *n (%)* |  |  |  |  |
| Difficult to access | 2 | (10%) | 3 | (12%) |
| Somewhat easy to access | 14 | (66%) | 12 | (48%) |
| Easy to access | 5 | (24%) | 10 | (40%) |
| **Trails for hiking**, *n (%)* |  |  |  |  |
| Difficult to access | 5 | (24%) | 3 | (12%) |
| Somewhat easy to access | 12 | (57%) | 15 | (60%) |
| Easy to access | 4 | (19%) | 7 | (28%) |
| **Home-based activity (playing outside)**, *n (%)* |  |  |  |  |
| No access | 1 | (5%) | 0 | (0%) |
| Difficult to access | 4 | (19%) | 3 | (12%) |
| Somewhat easy to access | 7 | (33%) | 8 | (32%) |
| Easy to access | 9 | (43%) | 14 | (56%) |
| **Community/family garden**, *n (%)* |  |  |  |  |
| No access | 1 | (5%) | 1 | (4%) |
| Difficult to access | 6 | (29%) | 4 | (16%) |
| Somewhat easy to access | 8 | (38%) | 16 | (64%) |
| Easy to access | 6 | (29%) | 4 | (16%) |
| **Water access for swimming**, *n (%)* |  |  |  |  |
| No access | 1 | (5%) | 0 | (0%) |
| Difficult to access | 2 | (9%) | 9 | (36%) |
| Somewhat easy to access | 12 | (57%) | 13 | (52%) |
| Easy to access | 6 | (29%) | 3 | (12%) |
| **Water access for water sports**, *n (%)* |  |  |  |  |
| No access | 1 | (5%) | 0 | (0%) |
| Difficult to access | 7 | (33%) | 4 | (16%) |
| Somewhat easy to access | 11 | (52%) | 20 | (80%) |
| Easy to access | 2 | (10%) | 1 | (4%) |
| **Water access for fishing**, *n (%)* |  |  |  |  |
| No access | 1 | (5%) | 1 | (4%) |
| Difficult to access | 7 | (33%) | 7 | (28%) |
| Somewhat easy to access | 11 | (52%) | 17 | (68%) |
| Easy to access | 2 | (10%) | 0 | (0%) |
